# Here to Stay: Biosimilars in Hematology

**DOI:** 10.1097/HS9.0000000000000323

**Published:** 2019-11-19

**Authors:** John Gribben, Giampaolo Merlini, Anton Hagenbeek

**Affiliations:** 1 Centre for Haemato-Oncology, Barts Cancer Institute, London, United Kingdom; 2 Amyloidosis Research and Treatment Center, Department of Molecular Medicine, University of Pavia, Pavia, Italy; 3 Department of Hematology, Amsterdam University Medical Centers, Amsterdam, The Netherlands

In the decade following the introduction of the first erythropoietin (2007) and G-CSF (2008) biosimilars on the European market, biosimilars have made their mark on hematology.^1^ The real breakthrough has come with the recent arrival of rituximab biosimilars, which have already achieved a considerable level of market access. Since its launch in 1997, rituximab, an anti-CD20 monoclonal antibody used for treating B-cell lymphomas, has significantly improved the life expectancy of lymphoma patients worldwide and plays an essential role in the development of less toxic but effective chemotherapy-free treatment regimens. Global rituximab sales reached a peak in 2016, totaling $8.58 billion,^2^ then fell to $7.3 billion in 2017 (of which $1.8 in Europe).^a^

EMA has so far authorized 2 rituximab biosimilars, under 6 different brand names with varying indications, and it has 2 more under review (April 2019). The first FDA approval of a rituximab biosimilar came in November 2018. Approvals by both regulators were based on phase III prospective randomized trials.

EHA welcomes the advance of biosimilars and the competition-boosting impact this has on the biologicals market. Biosimilars are equivalent to their reference products – the original, off-patent biological medicines to which they offer an alternative – in terms of safety and efficacy, but tend to be considerably less expensive. While uptake and price developments vary across countries, biosimilar (net) prices are often 20% to 30% or more below originator prices.^b^ By helping to drive down the prices of the reference biologics themselves, as well as across product classes, the overall pricing impact of biosimilars is significant and likely to increase. To the extent that they boost competition and reduce prices, thereby helping to increase patient access and relieve the pressure on healthcare budgets, EHA actively supports the acceptance and uptake of biosimilars.^3^

Increasing the uptake of biosimilars and the development of a sustainable biosimilars market require awareness and trust among professionals as well as patients. EHA unequivocally supports the prescription of properly assessed biosimilars – including those for rituximab – on condition that:

(1) their safety and efficacy are supported by solid clinical evidence (implicitly guaranteed by EMA once it approves a biosimilar for marketing authorization, including extrapolation of indications)
(2) information and education about the biosimilar medicine is of high quality and independent^c^
(3) biosimilar manufacturers introduce their drugs at fair and substantially reduced prices

With thorough marketing authorization procedures already in place (EMA), EHA will be contributing to realization of the second and third criteria by developing education tools for doctors, nurses, and patients and by engaging with biosimilar companies on price setting. Awareness-raising, knowledge-enhancing activities by EHA will include dedicated sessions at events, workshops and online education materials.

EHA's Task Force on Fair Pricing is actively encouraging manufacturers of rituximab biosimilars to help achieve the significant reduction in price levels that we believe is feasible. Through the combined effect of lower-cost biosimilars pushing down originator prices, an overall price reduction of 40% to 60% or more (vs the original reference biologics’ introduction prices) should be achievable in most cases. This would contribute substantially to enhancing the availability and accessibility of this most potent anti-lymphoma agent for patients.

Other stakeholders will also benefit from savings resulting from increased biosimilar uptake. Health systems will be able to spend freed-up funds on innovation, as Prof. Arnold Vulto argues in his article. This kicks off a *HemaSphere* series on biosimilars that, over the coming months, will present the views of various stakeholders. Hospitals can use savings to pay for other expensive innovative drugs to fulfill unmet needs in other categories of patients (with either malignant or benign hematological disorders) and stimulate innovation. A view from Eastern Europe will deal with the discrepancies in pricing and access issues in various parts of Europe. Biosimilar manufacturers will comment on the obstacles they face in gaining market access. Patients and nurses will be invited to share their perceptions and experiences, for instance with regard to safety, interchangeability and switching protocols. Last but not least, an article will be dedicated to the regulatory approach to biosimilars by the European Medicines Agency.

EHA's view is clearly a positive one: biosimilars that reduce treatment costs, improve patient access and free up funds for innovation are good for hematology.

## Footnotes

a IQVIA (via AV: IQVIA: Global sales 2017 7.3 billion US$, from which 1.8 billion in Europe).

b IQVIA, Advancing Biosimilar Sustainability in Europe. A Multi-Stakeholder Assessment, September 2018. IMS Health, The impact of biosimilar competition, June 2016.

c European Medicines Agency and the European Commission, Biosimilars in the EU: information guide for healthcare professionals. May 2017.
